# Incidence and risk of falls in patients treated for hematologic malignancies in the Intensive Hematology Unit

**DOI:** 10.1590/1518-8345.2953-3145

**Published:** 2019-04-29

**Authors:** Luz Alejandra Lorca, Cinara Sacomori, Valentina Paz Balagué-Ávila, Lorena Patricia Pino-Márquez, Fabiola Andrea Quiroz-Vidal, Leslie Ortega

**Affiliations:** 1 Hospital del Salvador, Servicio de Medicina Fisica y Rehabilitación, Santiago de Chile, RM, Chile; 2 Universidad Bernardo O´Higgins, Escuela de Kinesiología, Santiago de Chile, RM, Chile; 3 Centro de Referencia en Salud Cordillera Santiago, Servicio de Salud Metropolitano Oriente, Santiago de Chile, RM, Chile; 4 Hospital del Salvador, Unidad de Hematología Intensiva, Santiago de Chile, RM, Chile

**Keywords:** Falls, Hematological Neoplasms, Hospitalization, Risk, Care, Cancer, Quedas, Neoplasias Hematológicas, Hospitalização, Risco, Cuidados, Câncer, Caídas, Neoplasias Hematológicas, Hospitalización, Riesgo, Cuidados, Cáncer

## Abstract

**Objective:**

to determine the incidence and rate of risk of falls in adult patients treated for hematologic malignancies in the Intensive Hematology Unit of a reference hospital.

**Method:**

this is a retrospective observational study. A total of 101 patients were evaluated. The occurrence of falls was obtained from records of the unit and the predictive variables of the Hendrich II model were collected, namely: sex, presence of dizziness or vertigo, mental confusion, elimination problems, depression, use of benzodiazepines, use of anticonvulsants, and the Get up and Go test.

**Results:**

two fall events were reported in 101 patients (incidence of 1.98% over a 1.5-year period). Based on the cut-off point 5 of the Hendrich II Model, 30 patients (29.7%) were at risk of fall at the moment of hospital admission, 41 (40.6%) in the middle of the hospitalization period, and 38 (37.6%) at the moment of hospital discharge.

**Conclusions:**

patients treated for hematological malignancies presented low incidence and high risk of falls during hospitalization.

## Abstract (pt)

**Objetivo:**

determinar a incidência e a taxa de risco de quedas em pacientes adultos tratados por neoplasias hematológicas na Unidade de Hematologia Intensiva de um hospital de referência.

**Método:**

corresponde a um estudo observacional retrospectivo. Foram avaliados 101 pacientes. A ocorrência de quedas foi obtida a partir do registro da unidade e as variáveis preditivas do modelo Hendrich II foram coletadas: sexo, presença de tontura ou vertigem, confusão mental, problemas de eliminação, depressão, uso de benzodiazepínicos, uso de anticonvulsivantes e o teste *Get up and Go*.

**Resultados:**

dois eventos de quedas foram relatados em 101 pacientes (incidência de 1,98% em um período de 1,5 ano). Usando o ponto de corte cinco do Modelo Hendrich II, identificou-se que 30 pacientes (29,7%) apresentaram risco de queda no primeiro dia de hospitalização, 41 (40,6%) ao meio e 38 (37,6%) no momento da alta hospitalar.

**Conclusões:**

pacientes tratados por neoplasias hematológicas apresentaram baixa incidência e alto risco de quedas durante a hospitalização.

## Abstract (es)

**Objetivo:**

determinar la incidencia y la tasa de riesgo de caídas en pacientes adultos tratados por neoplasias hematológicas en la Unidad de Hematología Intensiva de un hospital de referencia.

**Método:**

corresponde a un estudio observacional retrospectivo. Se evaluaron 101 pacientes. La ocurrencia de caídas se obtuvo del registro de la unidad y las variables predictivas del modelo Hendrich II fueron recopiladas: sexo, presencia de mareos o vértigo, confusión mental, problemas de eliminación, depresión, uso de benzodiacepina, uso de anticonvulsionantes y la prueba *Get up and Go*.

**Resultados:**

dos eventos de caídas fueron reportados en 101 pacientes (incidencia de 1,98% en un período de 1,5 años). Utilizando el punto de corte 5 del Modelo Hendrich II, fue identificado que 30 pacientes (29,7%) tenían riesgo de caída al ingreso hospitalario, 41 (40,6%) en la mitad y 38 (37,6%) al egreso hospitalario.

**Conclusiones:**

los pacientes tratados por neoplasias hematológicas presentaron una incidencia baja y un alto riesgo de caídas durante la hospitalización.

## Introduction

Patient falls are still the most common of the adverse events reported in acute care centers, causing morbidity, mortality, fear of falling and prolonged loss of mobility, which may shorten people's life span^(^ ^1^ ^–^ ^2^ ^)^. Falls correspond to an event of high incidence in the hospital environment, with percentages between 1.1% and 22%, depending on the specificity of the patient^(^ ^3^ ^)^.

Three types of patient falls have been identified: (a) accidental (caused by the patient, such as slips or stumbling, usually attributed to some environmental hazard such as water on the floor); (b) anticipated physiological (falls of people considered at risk of falling); and (c) unanticipated physiological (falls attributed to physiological factors that cannot be predicted before the first fall)^(^ ^1^ ^)^. Physiological falls in hospitals account for 78% of the total falls, and they have been, therefore, the focus of many investigations attempting to identify factors associated with risk of falls^(^ ^4^ ^–^ ^5^ ^)^.

Patients hospitalized for cancer have even higher frequencies of falls, with a higher rate of injury, and greater severity when compared to hospitalized patients who do not have cancer^(^ ^2^ ^,^ ^6^ ^–^ ^9^ ^)^. About 23% to 42% of falls in patients hospitalized for cancer result in injuries, of which 2% to 9% end in severe events (fractures, subdural hematoma, excessive bleeding and death)^(^ ^7^ ^,^ ^9^ ^–^ ^11^ ^)^.

In this context, presence of diagnosis of cancer is already considered a risk factor for falls during hospitalization^(^ ^5^ ^)^. Younger patients admitted to oncology units, intensive care units, and units of infectious diseases are at increased risk of falls^(^ ^12^ ^)^. The cancer patients with the highest risk of falls during hospitalization are those with hematologic malignancies, brain cancer with presence of metastasis, those receiving transfusion of blood products, those under administration of chemotherapy, and those who had complications during the hospitalization period^(^ ^6^ ^)^. Another more recent study identified the use of technical help, history of falls, and presence of fatigue as risk factors among hospitalized cancer patients^(^ ^2^ ^)^.

In patients treated for hematologic malignancies, falls during periods of hospitalization may be related to: pathophysiological factors such as anemia and fatigue; effects of different treatments (chemotherapy, radiotherapy, transplantation of hematopoietic progenitors) such as diarrhea, vomiting, dizziness, muscle weakness, limited mobility; frequent changes in the state of health that characterize this population during hospitalization; and the manifestations of the disease^(^ ^13^ ^–^ ^15^ ^)^.

A significant number of investigations about falls have been conducted in elderly populations, whether in community-dwelling elderly or those in long stay institutions. However, there is little information on the incidence and risk of falls in the hospital environment among inpatients undergoing treatment of hematologic malignancies, which correspond mainly to young people. Knowing the incidence of falls and the related factors generates relevant information for care managers of hospitalized patients. To do this, it is necessary to use validated scales for the identification of risk of falls, such as the predictive Hendrich II Fall Risk Model, which is easy to use and previously applied to oncological populations^(^ ^6^ ^–^ ^7^ ^)^.

Thus, the objective of this study is to determine the incidence and the risk of falls in adult patients treated for hematologic malignancies during hospitalization at the Intensive Hematology Unit of a reference hospital between January 2016 and June 2017. It was hypothesized that patients hospitalized for treatment of hematological cancer would have a high incidence of falls and a high risk of falls, depending on aggressive treatments.

## Method

This was an observational study with a retrospective longitudinal design developed at the Intensive Hematology Unit of a reference hospital between January 2016 and June 2017. The Strengthening the Reporting of Observational Studies in Epidemiology (STROBE) guide was used to report observational studies^(^ ^16^ ^)^.

The present study was approved by the Ethics Committee and approved on 26/07/2016.

All clinical records of patients admitted for treatment of chemotherapy, chemo-radiotherapy or transplantation of hematopoietic progenitors at the Hospital Hematology Unit between January 2016 and June 2017, aged 18 years or older, with complete medical history and (daily) record of risk of falls were used. The exclusion criterion were: clinical records of patients with a history of hospitalization in the intensive hematology unit for apheresis.

Among a total of 158 cards, 101 met the eligibility criteria and were used in the study. In this study, using the predictive Hendrich II model, the risk assessments of falls made in the unit at the beginning, middle and end of the hospitalization period were considered.

Data on falls were collected through the revision of the Hospital Record of Falls, which contains a registry of all the events that happened in the Unit, including the falls, with a description of the situation and the place where they occurred.

Sociodemographic and clinical antecedents and information on the risk of falls were obtained from the review of 101 selected medical records with complete histories.

The literature presents some predictive models of falls for hospitalized patients, including the Morse scale (MFS), the fall risk scale (STRATIFY), the Cleveland Clinic Capone-Albert (CC-CA) and the predictive Hendrich II model^(^ ^4^ ^,^ ^17^ ^–^ ^19^ ^)^. Among these, only the last two were used for the oncological population.

For this study, the Hendrich II model was chosen because it includes criteria well established in the literature, validated and previously used in intensive hematology units. The Spanish version, which has undergone linguistic validation in Chile, was requested by e-mail to the authors. This instrument was chosen because it is quick and easy to apply and does not consider the bias of age, unlike other instruments commonly used to assess the risk of falls in health institutions. This model was built based on intrinsic factors of the physiological conditions of risk, excluding extrinsic factors. Furthermore, an important value is attributed to factors related to mobility^(^ ^4^ ^–^ ^5^ ^)^. This model also proved useful when applied in an emergency service to predict falls in the community and recurrences to the emergency service^(^ ^20^ ^)^.

The Hendrich II model contains eight predictors of risk of falls and can be used in several populations of acute patients. This model evaluates independent risk factors, namely: 1) Confusion, disorientation, impulsivity; 2) Symptomatic depression; 3) Altered eliminations; 4) Dizziness or vertigo; 5) Male gender; 6) Administration of antiepileptic drugs; 7) Administration of benzodiazepines; and 8) Low performance in the Get up and Go test^(^ ^4^ ^–^ ^5^ ^,^ ^7^ ^,^ ^21^ ^)^.

The final result corresponds to the sum of the individual scores of the factors above mentioned, the total score being 16. Scores of five or more are considered to indicate high risk of falls and require preventive measures^(^ ^4^ ^–^ ^5^ ^,^ ^7^ ^)^.

Patients who were categorized with scores equal to or greater than five according to the model received preventive strategies at the Unit, such as: installation of signaling at the entrance of the room, light on inside the room, bed with grills at the top, supervision of the steps in the room, supervision and use of appropriate footwear, paramedic care in the bathroom, installation of a portable bathroom inside the room if necessary, wearing of a ring bell, use of glasses or hearing aid if the patients were users of these devices, daily physical therapy care except weekends and holidays, as well as education directed to health personnel, patients and families.

Linguistic validation was performed in this study, in which 20 health professionals participated, including nurses and kinesiologists, led by an external bilingual health professional and whose native language was Spanish. The evaluators of risk of falls corresponded to a kinesiologist and a clinical nurse belonging to the intensive hematology unit. They were trained in the application of the evaluation instrument and later calibrated; the agreement was evaluated with the kappa index. The Kappa index of agreement between the two evaluators of the risk of falls (with and without risk of fall) was 100% (kappa = 1.0, p < 0.001). Considering each of the variables of the Hendrich II model, there was a 100% agreement in most of them, except for the Get up and Go test (kappa = 0.455, p = 0.017).

All data collected were organized in an Excel spreadsheet and later analyzed in the SSPS with descriptive statistics, such as absolute and relative frequency, mean and standard deviation. To verify the association between the occurrence of falls and the predictive variables, the chi-square test corrected with the Fisher's exact test was used. The Wilcoxon test was applied to compare the Hendrich II score for the risk of falls between the moment of hospital admission, the middle of the hospitalization period, and the end of the hospitalization period. A p < 0.05 was used in all tests. It was not possible to perform a regression analysis with the outcome falls because the number of fall events was very low.

## Results

The mean age of the patients was 35.6 (SD = 13.6) years. Table 1 presents the sociodemographic and clinical characterization of the participants, considering the general sample and the fall events. The majority of the participants were male (64.4%), the prevalent diagnosis was acute lymphoblastic leukemia (41.6%), and the majority were submitted to transplantation of hematopoietic stem cells (58.4%). The two women who suffered falls during the hospitalization period were treated with chemotherapy and radiotherapy, without transplantation.

**Table 1:** Sociodemographic and clinical characterization of the study participants of the sample in general and in relation to the fall events (n = 101), Santiago, Chile, RM, Chile, 2016-2017

		All patients (n=101)	Without fall event (n=99)	With fall event (n=2)
		n* (%)	n* (%)	n* (%)
Sex				
	Male	65 (64.4)	65 (65.7)	–
	Female	36 (35.6)	34 (34.3)	2 (100)
Marital status				
	Single	45 (44.5)	45 (45.5)	–
	Married/civil union	54 (53.5)	53 (53.5)	1 (50)
	Separated/divorced	2 (2.0)	1 (1.0)	1 (50)
Diagnosis				
	Acute Lymphoblastic Leukemia	42 (41.6)	41 (41.4)	1 (50)
	Acute Myeloid Leukemia	14 (13.9)	14 (14.1)	–
	Hodgkin's lymphoma	20 (19.8)	19 (19.2)	1 (50)
	Non-Hodgkin's Lymphoma	6 (5.9)	6 (6.1)	–
	Multiple myeloma	17 (16.8)	17 (17.2)	–
	Severe spinal aplasia	1 (1.0)	1 (1.0)	–
	Bulky lymphoma	1 (1.0)	1 (1.0)	–
Treatments				
	Chemotherapy	100 (99.0)	98 (99.0)	2 (100)
	Radiotherapy	32 (31.7)	30 (30.3)	2 (100)
	Immunotherapy	6 (5.9)	5 (5.1)	1 (50)
	Transplantation of hematopoietic progenitors	59 (58.4)	59 (58.6)	–
Type of transplant				
	Autologous	42 (41.6)	42 (42.4)	–
	Allogeneic haploidentical	11 (10.9)	11 (11.1)	–
	Identical allogeneic family donor	6 (5.9)	6 (6.1)	–
Comorbidities				
	Hypertension	8 (7.9)	8 (8.1)	–
	Diabetes Mellitus	3 (3.0)	3 (3.0)	–
Habits				
	Smoker	10 (9.9)	10 (10.1)	–
	Regular consumption of alcohol	17 (16.8)	17 (17.2)	–
	Drugs (marijuana/cocaine paste)	4 (4.0)	4 (4.0)	–

n* - number of participants (absolute frequency)

## Incidence and risk of falls

Two fall events were reported in 101 patients, with an incidence rate of 1.98% over a period of 1.5 years. The two events occurred at the time the patients performed hygienic activities with excretion in the portable bathroom in the bedroom and bathroom.

Table 2 presents the description of all variables of the Hendrich II Model to predict falls in hospitalized patients. It was observed that at the moment of hospital admission as well as in the middle and at the end of the hospitalization there was a low frequency of reports of mental confusion, depression and use of anticonvulsive medication among patients hospitalized for hematological cancer. In turn, most patients had problems with eliminations and used benzodiazepines. Dizziness was more frequent in the middle (30.7%) and at the end of hospitalization (28.7%) when compared to the moment of admission (21.8%). In the Get Up and Go Test, it was found that few patients could be alone in the first attempt at the moment of admission (37.6%) and that this frequency was still lower at the middle of the period of hospitalization (17.8%) and at hospital discharge (15.8%).

**Table 2:** Characterization of the criteria for risk of falls of the Hendrich II model at hospital admission, at the middle of the hospitalization period, and hospital discharge in the general sample and according to the presence of fall events (n = 101), Santiago, Chile, RM, Chile, 2016-2017

		Hospital admission			Middle of the hospitalization period			Hospital discharge		
		All patients (n=101)	No fall event (n=99)	With fall event (n=2)	All patients (n=101)	No fall event (n=99)	With fall event (n=2)	All (n=101)	No fall event (n=99)	With fall event (n=2)
		n* (%)	n* (%)	n* (%)	n* (%)	n* (%)	n* (%)	n* (%)	n* (%)	n* (%)
Mental confusion		0 (0)	0 (0)	0 (0)	1 (1.0)	1 (1.0)	0 (0)	1 (1.0)	1 (1.0)	0 (0)
Depression		6 (6.0)	6 (6.1)	0 (0)	7 (6.9)	7 (7.1)	0 (0)	4 (4.0)	4 (4.0)	0 (0)
Problem with eliminations		98 (97)	96 (97.0)	2 (100)	101 (100)	99 (100)	2 (100)	101 (100)	99 (100)	2 (100)
Dizziness		22 (21.8)	20 (20.2)	2 (100)	32 (30.7)	31 (31.3)	1 (50.0)	29 (28.7)	28 (28.3)	1 (50.0)
Male sex		65 (64.4)	65 (65.7)	0 (0)	66 (65.3)	65 (65.7)	1 (50.0)	66 (65.3)	66 (66.7)	0 (0)
Use of anti-convulsant medication		1 (1.0)	1 (1.0)	0 (0)	1 (1.0)	1 (1.0)	0 (0)	1 (1.0)	1 (1.0)	0 (0)
Use of benzodiazepines		95 (94.1)	93 (93.9)	2 (100)	99 (98.0)	97 (98.0)	2 (100)	98 (97.0)	96 (97.0)	2 (100)
Get up and Go Test – Get up from a chair										
	Get up in one movement	38 (37.6)	38 (38.4)	0 (0)	18 (17.8)	18 (18.2)	0 (0)	16 (15.8)	16 (16.2)	0 (0)
	Pushes and get up on the first attempt	49 (48.5)	48 (48.5)	1 (50.0)	59 (58.4)	58 (58.6)	1 (50.0)	62 (61.4)	61 (61.6)	1 (50.0)
	Able to get up after several attempts	9 (8.9)	8 (8.1)	1 (50.0)	19 (18.8)	18 (18.2)	1 (50.0)	18 (17.8)	17 (17.2)	1 (50.0)
	Unable to get up alone	5 (5.0)	5 (5.1)	0 (0)	5 (5.0)	5 (5.1)	0 (0)	5 (5.0)	5 (5.1)	0 (0)

n* - number of participants (absolute frequency)

Using the cut-off point of five from the Hendrich II Model, 30 patients (29.7%) were at risk at the moment of hospital admission, 41 patients (40.6%) in middle of the hospitalization, and 38 patients (37.6%) at hospital discharge.

A significant association was found between the presence of falls during hospitalization and the variable dizziness at hospital admission (x^2^ = 7.2, p = 0.047 corrected with Fisher's exact test). The other variables of the Hendrich II model were not associated with the presence of falls (p > 0.05).

A statistically significant difference was observed between the Hendrich II scores at the beginning and at the end of the hospitalization (p = 0.005) and between the beginning and the middle of the hospitalization period (p < 0.01). The median risk of falling at the moment of hospital admission [Median (Md) = 3; Interquartile interval (IR) = 2] was lower than that at the middle of the hospitalization period (Md = 4, IR = 2) and at hospital discharge (Md = 4, IR = 2).

## Discussion

It was found that patients treated for hematologic malignancies during hospitalization had a low incidence rate of falls (1.98%), which was within the percentages found in the international literature regarding hospitalized adult patients (1.1% - 22.0%)^(^ ^1^ ^,^ ^22^ ^)^. It is possible that this low incidence rate is due to the fact that the patients were mainly young people (mean age 35.6 years). Also, in the Intensive Hematology Unit in this study, traditional measures of fall prevention were used by the nursing team, including: keeping the bars up, keeping the room well lit, using a ring bell, wearing of proper shoes, supervising the floors, assisting the patient during the shower, using signals at the entrance of the room, and daily evaluation of the risk of falls. Furthermore, patients were seen at daily basis by a kinesiologist (physical therapist) who performed education activities to patients and their families, and a daily exercise routine to promote change of positions and transfers, mobility, balance and stability training exercises that favor independence in everyday activities, in accordance with international recommendations^(^ ^7^ ^,^ ^23^ ^–^ ^24^ ^)^. Thus, the daily assessment of the risk of falls in patients under treatment for hematologic cancer is recommended to identify patients at risk and to implement appropriate prevention measures with a multidisciplinary approach^(^ ^25^ ^)^. The low number of adverse events, such as in-hospital falls is an indicator of the quality of the nursing service^(^ ^2^ ^)^.

Falls are a very important issue in the health of hospitals. Despite global efforts to prevent falls, there has been an increase in the number of patient falls in hospitals and other health services. This increase can be explained by the increase in the recording systems of these patients, in the number of elderly patients, patients more severely affected by diseases, and patients with higher impairments, including patients with high sedation and shorter time spent by the nursing staff at the patient's bedside^(^ ^26^ ^)^. It was shown that cancer patients suffering from fall events have a higher risk of dying compared to non-cancer patients who suffer falls (odds ratio = 2.58, 95% CI: 1.91-3.49)^(^ ^27^ ^)^.

When considering the predictive Hendrich II model for the evaluation of risk of falls, this study identified that people treated for hematologic malignancies presented a higher prevalence of risk of falling in the middle of the hospitalization period (40.6%) compared to admission (29.7%) and hospital discharge (37.6%). These results are similar to those obtained in another study^(^ ^22^ ^)^. This can be explained by aggressive treatments that patients receive, such as chemotherapy for induction, consolidation or transplantation of hematopoietic progenitors, which produce important symptoms, including fatigue^(^ ^28^ ^)^, mucositis, nausea, diarrhea and vomiting, pain and lack of appetite^(^ ^29^ ^)^. In addition, infections that produce febrile neutropenia (prevalence around 40%), a condition that prolongs hospitalization, delays new cycles of chemotherapy, and even increases mortality, is common in these patients^(^ ^30^ ^)^.

In this study, a significant association between the presence of dizziness at hospital admission and the occurrence of falls during hospitalization was identified. In addition, dizziness was found to be more present in the middle (30.7%) and at the end of hospitalization (28.7%), when compared to hospital admission (21.8%). Dizziness has been identified as a risk factor associated with falls in the hospital context^(^ ^31^ ^)^. In patients treated for hematologic malignancies during hospitalization, dizziness is a symptom that may be present and be produced by multiple factors, such as: side effects of chemotherapy in the central nervous system (vinca alkaloids, cytarabine and methotrexate), orthostatic hypotension, fatigue, anemia and use of drugs such as benzodiazepines^(^ ^4^ ^,^ ^7^ ^,^ ^15^ ^,^ ^32^ ^–^ ^36^ ^)^.

It was observed that both at hospital admission and in the middle and end of hospitalization, there was a low frequency of reports of mental confusion, depression and use of anticonvulsant medication among patients hospitalized for hematologic cancer. Also, it was identified that the majority of the patients presented problems with eliminations and used benzodiazepines. The use of drugs such as benzodiazepines, anticonvulsants, corticosteroids and antidepressants puts patients treated for hematologic cancer at a greater risk of falls during hospitalization^(^ ^36^ ^–^ ^37^ ^)^. A recent study of 768 people hospitalized with cancer in China found that the use of drugs that affect the central nervous system (hypnotics, sedatives, serotonin inhibitors, opioids, antiepileptics and antipsychotics) increased by 4.29-fold the likelihood of suffering a fall event^(^ ^38^ ^)^.

The risk of falling in younger patients hospitalized for cancer may be associated with recurrent elimination needs^(^ ^8^ ^,^ ^11^ ^)^. These patients usually present urinary incontinence, diarrhea or urinary urgency, and these things can significantly increase the possibility of falls, as they use to occur mainly near the patient's bed, in the bathroom or in the corridor^(^ ^23^ ^,^ ^39^ ^)^.

Regarding the place of the falls, in the present study, it was identified that the two events occurred in the bedroom and in the bathroom during hygiene of excretion activities. This agrees with other studies that indicate the room as the place with the highest frequency of falls (80.4%), followed by the bathroom (17.1%)^(^ ^23^ ^,^ ^26^ ^,^ ^39^ ^)^.

Regarding the mobility evaluated with the Get up and Go test, a few patients were able to stand up alone in the first attempt at hospital admission (37.6%) and this frequency was even lower at the middle of the hospitalization (17.8%), and at hospital discharge (15.8%). A retrospective study of onco-hematologic patients showed that falls were strongly associated with mobility deficit and loss of independence in daily activities^(^ ^32^ ^)^. In particular, patients who undergo transplantation of hematopoietic progenitors are pretreated with chemotherapy, and during the course of hospitalization, they receive more chemotherapy with a high potential to cause peripheral neuropathy, which can lead to muscle weakness with motor function and walking^(^ ^32^ ^,^ ^40^ ^)^. Patients undergoing allogeneic transplantation, those with host-to-graft disease, as the first line agent are usually treated with high doses of corticosteroids for prolonged periods, which have been associated with myopathy and increased risk of falls^(^ ^41^ ^–^ ^42^ ^)^.

In this context, it is suggested that, for the prevention of risk of falls, besides traditional measures, the nursing teams in intensive hematology units integrate other strategies that complement the care plans by other professionals, such as occupational therapists and kinesiologists (physical therapists), promoting interventions to maintain the motor skills necessary to perform the activities of daily living^(^ ^24^ ^,^ ^43^ ^)^. A study of nurses in a hospital identified that among the fall prevention activities, the most frequently applied were: identification of cognitive deficits and risk factors, and caring for mobility and transfer of patients^(^ ^44^ ^)^. On the other hand, activities related to infrastructure, such as the use of guardrails, alarm systems and call lighting, are carried out on a smaller scale^(^ ^44^ ^)^. Some technological tools, such as motion sensors, that detect when patients get out of bed and there is risk of falling, which have been tested in hospitals and found to be useful^(^ ^45^ ^)^.

Although patients with hematologic and cerebral cancer have been identified as the oncology population most vulnerable to falls in the hospital context^(^ ^6^ ^)^, there is still little information on the predictive factors of falls and prevention strategies for people treated for hematologic malignancies. The predictive Hendrich II model evaluates a significant number of factors representative of a single population in a hospital context, such as patients treated for hematologic malignancies. However, the gender factor considered in this model was not a good predictive indicator to evaluate the risk of falls in this population, because patients who fell were female, contrary to what the model predicts.

This study has as strong point the fact that it was conducted at the national referral center for treatment of hematologic malignancies, with a very specific population, which is treated with international standards of care. It was a pioneering study in our country that contributes to a better knowledge of the population treated for hematologic malignancies and of the use of a tool for evaluation of risk of falls that is more specific for this population, unique in the hospital context.

Through the results obtained in this study, we can suggest the development of further researches that promote the use of instruments for the evaluation of the risk of falls with greater specificity according to the population to be evaluated, as it is the case of patients hospitalized for treatment of hematologic neoplasias, which correspond mainly to young people.

This study has the limitation of having been performed in a single hospital. Thus, our results cannot be generalized. Furthermore, the low incidence of falls prevented statistical tests, such as logistic regression, to confirm whether the Hendrich II model was valid for this population.

## Conclusion

The incidence rate identified in this study (1.98%), although seemingly low, suggests the need for greater attention from the health team to strengthen the planning of prevention strategies with an interdisciplinary approach that promotes patient safety.

In this study, it was found that a significant percentage of people hospitalized for treatment of hematologic malignancies had a high risk of falling according to the predictive Hendrich II model, either at hospital admission (29.7%), in the middle of the hospitalization period (40.6%), or hospital at discharge (37.6%).
